# Bioactivity Research in *Ganoderma lucidum*: A Scientometric Analysis of Global Trends, Translational Gaps, and Emerging Research Frontiers

**DOI:** 10.3390/biology15141100

**Published:** 2026-07-08

**Authors:** Christian Joseph N. Ong, Jamil Allen G. Fortaleza, Jowi Tsidkenu Pili Cruz, Edison D. Ramos, Joel G. Matamis, Janice Kaylyn Lonogan, Amelda C. Libres, Carina M. Magtibay, Jose Edwardo R. Mamaat, Maylaine M. de Leon, Carlos S. de Leon, Jose Jurel M. Nuevo, Rich Milton R. Dulay

**Affiliations:** 1 Department of Biology, College of Science, De La Salle University, Manila 1004, Philippines; christian_joseph_ong@dlsu.edu.ph (C.J.N.O.); jowi.cruz@dlsu.edu.ph (J.T.P.C.); richmiltondulay@clsu.edu.ph (R.M.R.D.); 2 National University, Sampaloc, Manila 1008, Philippines; edramos@national-u.edu.ph; 3 School of Medical Laboratory Sciences, St. Dominic College of Asia, Bacoor 4102, Philippines; jgmatamis@sdca.edu.ph; 4 Office of the Vice President for Academic Affairs, University of Baguio, Baguio 2600, Philippines; janub95@e.ubaguio.edu; 5 College of Medical Laboratory Science, Liceo de Cagayan University, Cagayan de Oro City 9000, Philippines; alibres@liceo.edu.ph; 6 College of Allied Medical Professions, Lyceum of the Philippines University, Batangas 4200, Philippines; cmmagtibay@lpubatangas.edu.ph; 7 Department of Medical Technology, Far Eastern University, Manila 1008, Philippines; jemamaat@feu.edu.ph; 8 Department of Medical Laboratory Science, Dr. Carlos S. Lanting College, Quezon City 1116, Philippines; maydeleonrmt88@gmail.com (M.M.d.L.); mls.mt94@gmail.com (C.S.d.L.); 9 College of Medical Laboratory Science, Our Lady of Fatima University, Valenzuela City 1440, Philippines; jmnuevo@fatima.edu.ph; 10 Center for Tropical Mushroom Research and Development, Central Luzon State University, Science City of Muñoz 3120, Philippines

**Keywords:** *Ganoderma lucidum*, scientometric analysis, bibliometrics, bioactivity, medicinal mushrooms

## Abstract

*Ganoderma lucidum* (Lingzhi or Reishi) is among the most extensively studied medicinal mushrooms globally, owing to its diverse bioactive compounds and broad pharmacological properties. A scientometric analysis using the Scopus database was conducted to investigate global research trends on the bioactivity of *G. lucidum*. Bibliometric indicators, collaboration networks, keyword co-occurrence, thematic evolution, conceptual structure mapping, and life cycle modeling were assessed using Bibliometrix/Biblioshiny and VOSviewer version 1.6.20. A total of 2877 publications were included in the analysis. Scientific output increased markedly after 2010, peaking in 2025, indicating sustained growth in the field. China was identified as the leading contributor in terms of publication volume, influential authors, and prominent institutions, while international collaboration networks showed expanding global participation. Keyword and thematic analyses demonstrated a shift from early research focused on bioactivity screening, polysaccharides, and cultivation to mechanistic and translational investigations involving apoptosis, oxidative stress, immunomodulation, metabolomics, gut microbiota, network pharmacology, and disease-specific applications. Life cycle analysis suggested that the field is still in a rapid growth phase and has not yet reached saturation. Research on *G. lucidum* has evolved into a mature and highly interdisciplinary field, characterized by increasing scientific productivity, expanding international collaboration, and a growing emphasis on mechanistic and translational studies.

## Simple Summary

In recent decades, research on the bioactive compounds of *Ganoderma lucidum* has expanded rapidly across multiple scientific disciplines. The increasing volume of publications has complicated the identification of major research trends, influential contributors, and emerging directions within the field. This scientometric analysis evaluated 2877 research articles indexed in Scopus to present a comprehensive overview of global research on the bioactivity of *G. lucidum*. The results indicate a substantial increase in scientific output; significant contributions from Asian countries, particularly China; and a transition from basic bioactivity screening to mechanistic and translational studies involving metabolomics, gut microbiota, immunomodulation, and disease-specific applications. These findings offer researchers, clinicians, and industry stakeholders a clearer understanding of the current research landscape and highlight promising areas for future investigation and therapeutic development.

## 1. Introduction

*Ganoderma lucidum* (Curtis) P. Karst., commonly referred to as Lingzhi or Reishi, is one of the most extensively studied medicinal mushrooms worldwide and has been used in traditional Asian medicine for more than 2000 years. Due to its health-promoting properties, *G. lucidum* has attracted considerable scientific attention for its diverse bioactive metabolites and broad therapeutic potential. Recent pharmacological investigations have confirmed that this mushroom contains numerous biologically active constituents, including polysaccharides, triterpenoids, proteins, peptides, sterols, phenolic compounds, and nucleotides, each contributing to its wide-ranging biological effects [1,2]. The combination of traditional medicinal knowledge and modern biomedical research has established *G. lucidum* as a prominent model organism in natural product discovery, pharmacology, biotechnology, and functional food research [3].

Although *G. lucidum* remains the most widely recognized scientific name in the medicinal mushroom literature, its taxonomy has undergone substantial revision over the past two decades. Molecular phylogenetic studies have demonstrated that the true European *G. lucidum* is distinct from several morphologically similar Asian taxa that were historically identified under the same name. In particular, the medicinal “Lingzhi” cultivated and investigated throughout East Asia is now widely recognized as *G. lingzhi*, while other closely related species, including *G. sichuanense*, have also contributed to the historical use of the name *G. lucidum* in scientific publications [4,5,6]. Consequently, a considerable proportion of the earlier literature indexed under *G. lucidum* likely encompasses multiple phylogenetically distinct species within the *G. lucidum* species complex.

The pharmacological versatility of *G. lucidum* is largely attributed to its polysaccharides and triterpenoids, which are among the most extensively characterized bioactive compounds from this mushroom. Both experimental and clinical studies have demonstrated antioxidant, anti-inflammatory, immunomodulatory, antimicrobial, antidiabetic, hepatoprotective, neuroprotective, cardioprotective, and anticancer activities associated with these metabolites [7,8]. Furthermore, growing evidence suggests that bioactive compounds from *G. lucidum* modulate various molecular pathways involved in immune responses, oxidative stress reduction, apoptosis, inflammation, and cellular signaling, thereby increasing its therapeutic potential [9,10]. These results have stimulated significant interest in exploring *G. lucidum* as a source of novel pharmaceuticals, nutraceuticals, and functional food ingredients.

Advancements in analytical chemistry, metabolomics, molecular biology, and biotechnology have enabled the identification of novel compounds and elucidated the mechanisms underlying the bioactivity of *G. lucidum*. Current research encompasses not only polysaccharides and triterpenoids but also proteins, meroterpenoids, sterols, fatty acids, and other secondary metabolites with potential biomedical applications [2,3]. Consequently, scientific publications on the bioactivity of *G. lucidum* have increased substantially over the past two decades, highlighting its growing significance in pharmaceutical, biomedical, agricultural, and functional food sciences [9]. However, the expanding literature is dispersed across multiple disciplines, complicating comprehensive assessment of the field’s intellectual structure, principal contributors, and emerging research directions.

Bibliometric and scientometric analyses provide quantitative tools for evaluating the development of scientific fields by measuring publication output, citation metrics, collaboration networks, and thematic evolution. These methods are increasingly used to map research landscapes, identify knowledge gaps, and highlight emerging trends in biomedical and natural product sciences [11]. Although numerous narrative and systematic reviews have examined the chemical composition, pharmacological properties, and therapeutic applications of *G. lucidum* [1,7]. Given the rapid growth and interdisciplinary nature of the field, a systematic evaluation of global research patterns is essential to clarify its development and future trajectory.

Therefore, this study aims to conduct a scientometric analysis of global research on the bioactivity of *G. lucidum*. Specifically, the study seeks to (1) evaluate publication productivity and citation trends over time; (2) identify the most influential authors, institutions, countries, and journals; (3) examine international collaboration networks and patterns of scientific cooperation; (4) determine major research hotspots and thematic clusters associated with the bioactivity of *G. lucidum*; and (5) explore emerging themes and future research directions.

## 2. Materials and Methods

### 2.1. Study Design

This study employed a scientometric approach to examine the global research landscape of *G. lucidum* bioactivity. Bibliographic metadata were analyzed to evaluate publication trends, citation performance, collaboration networks, and thematic evolution, providing a quantitative overview of the field’s development and emerging research directions.

### 2.2. Data Source

Bibliographic records were retrieved exclusively from the Scopus database because of its broad coverage of peer-reviewed journals and standardized bibliographic metadata, making it well suited for scientometric analysis. Metadata included publication year, authors, affiliations, journal source, abstracts, keywords, subject categories, and citation counts. Using a single database ensured consistent metadata and citation indexing while minimizing duplicate records and discrepancies that arise when combining multiple databases. However, publications indexed only in regional or non-Scopus databases may not be represented.

### 2.3. Search Strategy and Data Extraction

**Figure 1:**
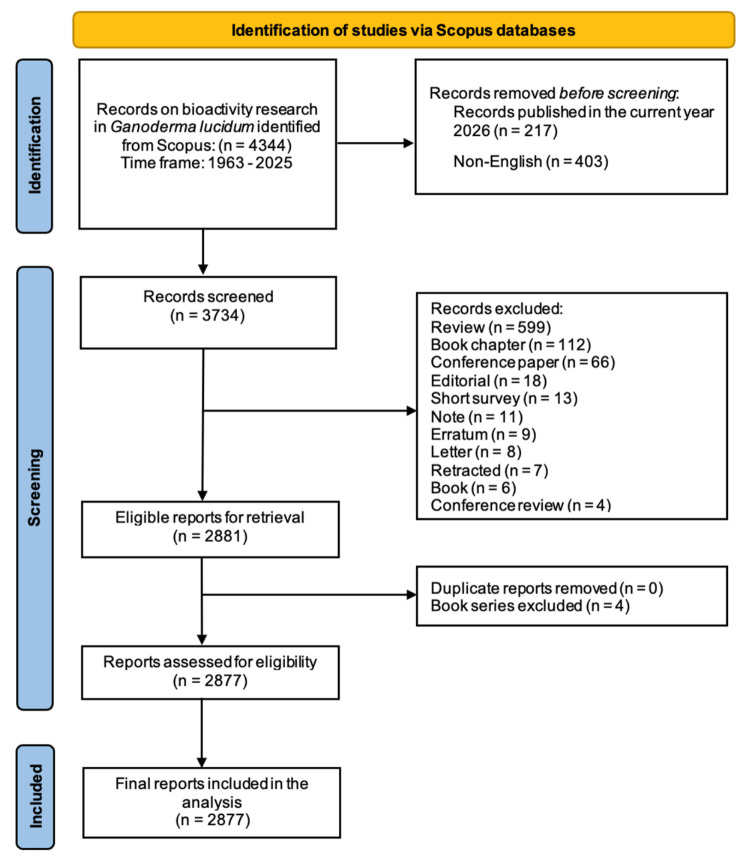
PRISMA flowchart diagram of the scientometric study on bioactivity research on *G. lucidum*.

A systematic search was conducted in Scopus on 14 June 2026, using the TITLE-ABS-KEY field to identify publications related to the bioactivity of *G. lucidum*. The search incorporated the scientific name, common names, and bioactivity-related keywords to maximize retrieval of relevant studies while maintaining specificity. The study selection process followed a PRISMA-based workflow, including identification, screening, eligibility assessment, and final inclusion of records for scientometric analysis (Figure 1).

The search strategy combined the scientific name (*Ganoderma lucidum*), commonly used synonyms (e.g., Lingzhi, Ling Zhi, and Reishi), and keywords describing biological activities, pharmacological properties, therapeutic applications, and mechanistic investigations. Search terms were applied to the TITLE-ABS-KEY field to maximize retrieval of relevant publications while minimizing unrelated records. Activity-related descriptors included antioxidant, antimicrobial, anti-inflammatory, anticancer, immunomodulatory, hepatoprotective, neuroprotective, cardioprotective, antidiabetic, and other commonly reported bioactivities, together with methodological terms such as in vitro, in vivo, and mechanism of action.

To ensure reliable bibliometric and citation analyses, publications indexed in 2026 were excluded because citation accumulation and database indexing were incomplete at the time of data retrieval. The analysis was restricted to English-language, peer-reviewed original research articles, while reviews, editorials, letters, conference papers, notes, book chapters, and other non-research publications were excluded to maintain dataset consistency. Eligible records were exported from Scopus in CSV format, including complete bibliographic metadata, for preprocessing and scientometric analysis. Journal CiteScore indicators were also retrieved from Scopus to contextualize the scholarly influence of publication sources.

The complete search string utilized in this study is provided below:

TITLE-ABS-KEY (“*Ganoderma lucidum*” OR “Lingzhi” OR “Ling Zhi” OR “Reishi”) AND TITLE-ABS-KEY (“bioactivity” OR “biological activity” OR pharmacological OR therapeutic OR medicinal OR antimicrobial OR antibacterial OR antifungal OR antiviral OR antioxidant OR anti-inflammatory OR anticancer OR antitumor OR cytotoxic OR immunomodulatory OR immunological OR hepatoprotective OR neuroprotective OR cardioprotective OR antidiabetic OR hypoglycemic OR antihyperglycemic OR antiobesity OR anti-aging OR “free radical scavenging” OR quorum-quenching OR “quorum sensing inhibition” OR “therapeutic potential” OR “in vitro” OR “in vivo” OR “mechanism of action”) AND (EXCLUDE (PUBYEAR, “2026”)) AND (LIMIT-TO (LANGUAGE, “English”)) AND (LIMIT-TO (DOCTYPE, “ar”)) AND (LIMIT-TO (SRCTYPE, “j”)).

### 2.4. Data Cleaning and Harmonization

The retrieved records were manually curated to improve the consistency and reliability of the bibliometric dataset. Duplicate records were removed, while author names, institutional affiliations, and keywords were standardized using a manually developed thesaurus to merge synonymous terms, spelling variants, abbreviations, and lexical inconsistencies. Generic indexing terms and irrelevant keywords were excluded to reduce analytical noise. Minimum thresholds of 5 keyword occurrences and 10 institutional publications were applied to improve the clarity and interpretability of network visualizations. Titles and abstracts were also screened to exclude records not directly related to the bioactivity of *G. lucidum*.

Keyword harmonization involved consolidating synonymous and conceptually equivalent terms into standardized descriptors. For example, “*Ganoderma lucidum*”, “*G. lucidum*”, “reishi”, and “lingzhi” were unified under a single term, while related expressions such as “antioxidant activity” and “antioxidant properties”, “anticancer activity” and “antitumor activity”, and “medicinal mushroom” and “medicinal fungi” were merged to improve the accuracy and consistency of thematic analyses.

### 2.5. Data Analysis

**Figure 2:**
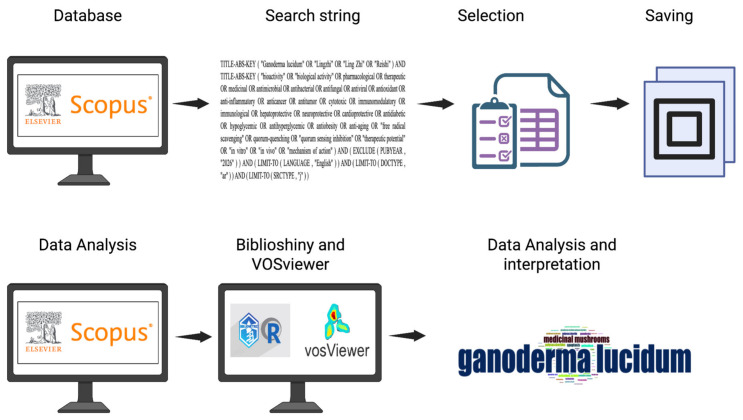
Schematic workflow of the scientometric analysis conducted in this study, illustrating the processes of database retrieval from Scopus, search strategy formulation, study selection and data exportation, followed by enrichment and science mapping analyses using Bibliometrix/Biblioshiny (R package) and VOSviewer for visualization.

Bibliometric analyses were performed using the Bibliometrix package version 5.0 and its Biblioshiny interface in R, while network visualization was conducted using VOSviewer version 1.6.20 (Universiteit Leiden and CWTS Meaningful Metrics). Bibliometrix was used to evaluate publication output, citation performance, source productivity, institutional contributions, and temporal research trends, whereas VOSviewer generated co-authorship, institutional, country, and keyword co-occurrence networks. Science mapping analyses, including thematic evolution, strategic thematic mapping, multidimensional scaling (MDS), and temporal overlay visualization, were performed to characterize the intellectual structure and evolution of *G. lucidum* bioactivity research [12,13]. The overall analytical workflow is presented in Figure 2.

## 3. Results

### 3.1. Annual Publication, Citation Trends, and Temporal Dynamics

**Figure 3:**
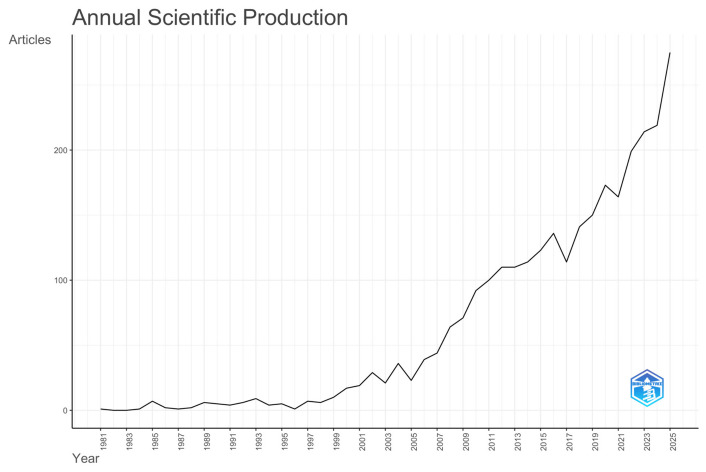
Trend analysis of global annual scientific publications on bioactivity research on *G. lucidum*.

A total of 2877 original research articles were retrieved and analyzed from the Scopus database. The trend analysis indicates that global scientific production on the bioactivity of *G. lucidum* remained relatively limited from 1981 to the late 1990s, with annual outputs generally fluctuating at very low levels (Figure 3). This early phase suggests that bioactivity research on *G. lucidum* was still emerging, likely concentrated in descriptive pharmacological studies and traditional medicinal investigations. A gradual increase became evident after 2000, followed by a more pronounced expansion from approximately 2007 onward, when annual publications rose steadily from fewer than 50 articles to nearly 100 by 2010. This pattern reflects the transition of *G. lucidum* research from a niche medicinal mushroom topic into a more established field within natural products, pharmacology, biotechnology, and biomedical sciences.

A strong acceleration in scientific production became evident after 2010, with annual output exceeding 100 publications and continuing to rise despite minor fluctuations between 2016 and 2020. Publication activity rose further after 2021, reaching nearly 275 articles in 2025. This sustained upward trend reflects growing global interest in the bioactivity of *G. lucidum* and its therapeutic potential. The continued increase in research output also highlights the field’s expanding interdisciplinary nature, driven by advances in molecular biology, metabolomics, pharmacological screening, and functional food research.

**Figure 4:**
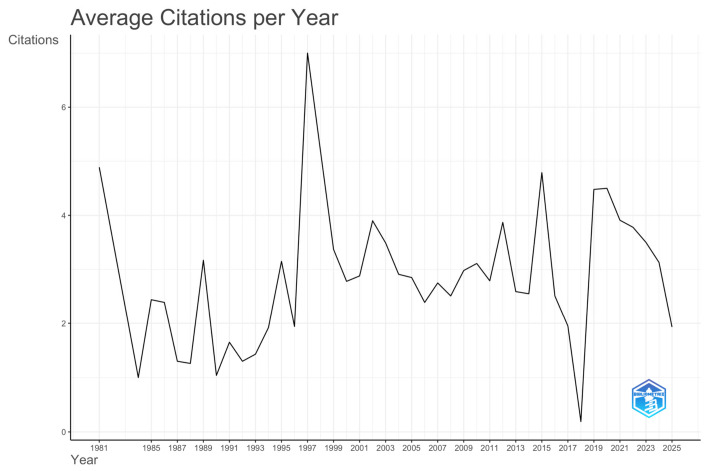
Citation trend analysis of bioactivity research on *G. lucidum*.

The citation trend shows a highly fluctuating pattern in the average annual citations of bioactivity research on *G. lucidum*, indicating uneven scholarly impact across publication years (Figure 4). Early outputs from the 1980s and 1990s display marked variability, which is expected in periods with low annual publication volume, where a small number of highly cited articles can strongly influence yearly citation averages.

The most prominent citation peak occurred around 1997, reaching more than seven average citations per year, suggesting that publications from this period encompassed several landmark studies that established the pharmacological foundation of *Ganoderma lucidum* research. These pioneering investigations systematically demonstrated the immunomodulatory, antitumor, antioxidant, hepatoprotective, and other therapeutic activities of *Ganoderma* polysaccharides and triterpenoids while simultaneously providing comprehensive insights into their chemical composition, structural characteristics, and pharmacological potential. Collectively, these studies transformed *G. lucidum* from a medicinal mushroom primarily recognized in traditional Asian medicine into an internationally acknowledged source of bioactive natural products with broad pharmaceutical and nutraceutical applications. They also laid the scientific foundation for subsequent mechanistic investigations exploring cellular signaling pathways, immune regulation, oxidative stress modulation, and apoptosis. Consequently, the sustained citation performance observed over the following decades reflects the enduring influence of these seminal publications, which established polysaccharides and triterpenoids as the principal bioactive constituents of *G. lucidum* and catalyzed extensive research into their molecular mechanisms, therapeutic efficacy, and translational biomedical applications.

From the 2000s onward, citation performance became more stable, generally ranging between two and four citations per year, reflecting the maturation and expansion of the research field. Notable increases around 2002, 2012, 2015, and 2019–2020 suggest renewed scholarly attention linked to advances in pharmacological screening, molecular mechanism studies, and biomedical applications of *G. lucidum*. The decline after 2021, particularly toward 2025, should be interpreted cautiously because recent publications have had less time to accumulate citations. Thus, the apparent reduction in citation averages in recent years reflects citation-window effects rather than diminished scientific relevance.

**Figure 5:**
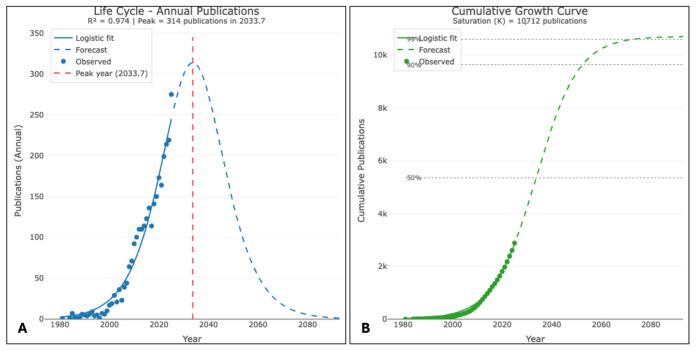
Life cycle of global annual scientific production (**A**) and the cumulative growth curve (**B**) on bioactivity research on *G. lucidum*.

The life cycle model of annual scientific production (Figure 5A) shows that bioactivity research on *G. lucidum* is currently in a rapid growth phase, as indicated by the close alignment between the observed publication counts and the fitted logistic curve. The high model fit (R^2^ = 0.974) suggests that the publication trajectory exhibits strong growth, with annual output increasing sharply after the early 2000s and accelerating further after 2010. The model forecasts a peak annual production of approximately 314 publications around 2033.7, indicating that the field has not yet reached maximum productivity. This pattern reflects the sustained expansion of research on *G. lucidum* bioactivity, driven by growing scientific interest and ongoing advances in pharmacological, molecular, and translational investigations.

The cumulative growth curve (Figure 5B) further supports the interpretation that research on *G. lucidum* bioactivity remains in an expansion stage rather than a mature or saturated phase. The logistic projection estimates a saturation level of approximately 10,712 cumulative publications, with the curve expected to approach 50%, 90%, and 99% of total projected output across future decades. The steep projected rise between the 2030s and 2050s suggests continued growth in research productivity before eventual stabilization. The current observed cumulative output remains well below the projected saturation threshold, indicating substantial remaining capacity for knowledge production, thematic diversification, and methodological advancement within the field.

### 3.2. Geographical Distribution of Scientific Publications

**Figure 6:**
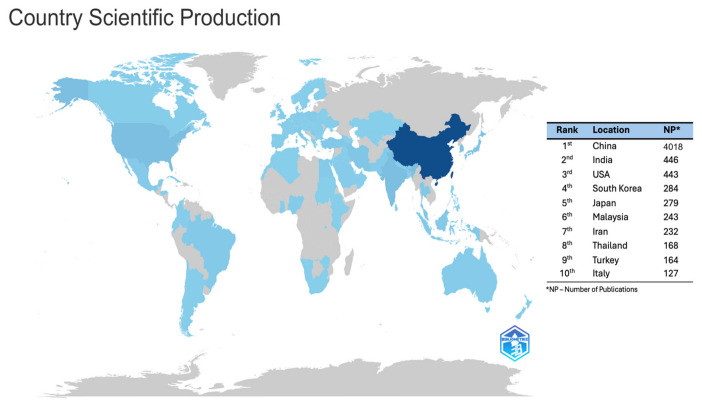
Geographical distribution of scientific production on bioactivity research on *G. lucidum*.

Location-specific scientific output shows a highly uneven global distribution of bioactivity research on *G. lucidum*, with China overwhelmingly dominating the field, accounting for 4018 publications (Figure 6). This far exceeds the next most productive countries, including India (n = 446), the United States (n = 443), South Korea (n = 284), Japan (n = 279), Malaysia (n = 243), Iran (n = 232), Thailand (n = 168), Turkey (n = 164), and Italy (n = 127). The darkest shading over China in the map confirms its central role as the principal producer of *G. lucidum* bioactivity research, likely reflecting its long-standing medicinal use of Lingzhi, extensive fungal biotechnology infrastructure, and strong research investment in natural products, pharmacology, and traditional medicine.

Beyond China, the map and dataset indicate broad but comparatively lower participation across Asia, North America, Europe, South America, Africa, and Oceania. Asian countries dominate the upper tier, particularly India, South Korea, Japan, Malaysia, Iran, and Thailand, suggesting that regional familiarity with medicinal mushrooms strongly influences research productivity. Moderate output from the United States, Italy, Brazil, Serbia, Nigeria, Poland, Mexico, Pakistan, Egypt, Spain, and Australia demonstrates the field’s international diffusion, while many countries contribute only a small number of publications. This pattern indicates that although research on G. lucidum bioactivity has achieved global reach, scientific output remains concentrated in a limited number of countries, with Asia serving as the main geographic center of knowledge generation.

### 3.3. Most Relevant Authors

The table shows that the most productive authors in *G. lucidum* bioactivity research are heavily concentrated in Asia, particularly China (Table 1). Zhong, Jian-Jiang ranked first with 47 publications, followed by Zhao, Ming Wen with 38, Lin, Zhi-Bin with 37, Ren, Ang with 34, and Shi, Liang with 33. This distribution indicates that Chinese scholars hold leading positions in the field, reflecting a strong scientific infrastructure, ethnomedicinal relevance, and sustained national research interest in *G. lucidum* as a medicinal mushroom.

Institutionally, the leading contributors are affiliated with major Chinese universities and research centers, including Shanghai Jiao Tong University, Nanjing Agricultural University, Peking University Health Science Center, Shanghai Academy of Agricultural Sciences, Zhejiang University, and Guangdong Yuewei Edible Fungi Technology Co., Ltd. The presence of Shimizu, Kuniyoshi from Japan and Janardhanan, Krishnan Krishnankutty from India shows that influential contributions also extend to other Asian research systems, although China remains the dominant contributor. This authorship pattern suggests that bioactivity research on *G. lucidum* is shaped largely by regional expertise in medicinal fungi, natural products, agricultural biotechnology, pharmacology, and traditional medicine.

### 3.4. Most Relevant Sources

The top journals indicate that bioactivity research on *G. lucidum* is published across specialized medicinal-mushroom, natural-product, pharmacology, and biomacromolecular science outlets (Table 2). The International Journal of Medicinal Mushrooms ranked first by productivity with 258 publications and 4734 citations, confirming its role as the principal publication venue for medicinal fungi research. However, journals such as the International Journal of Biological Macromolecules, Journal of Ethnopharmacology, and Carbohydrate Polymers show stronger citation efficiency, with higher average citations per paper and h-index values, reflecting the high impact of studies on polysaccharides, bioactive macromolecules, ethnopharmacology, and carbohydrate-based compounds.

The distribution of sources also demonstrates the interdisciplinary nature of *G. lucidum* bioactivity research. High-output journals such as *Molecules*, *Frontiers in Pharmacology*, *Scientific Reports*, and *PLOS One* suggest broad dissemination across open-access and multidisciplinary platforms, while *Phytochemistry* recorded the highest average citation per paper, indicating strong influence despite a smaller publication volume. The presence of publishers such as Elsevier, Springer Nature, MDPI, Frontiers, Wiley, and Begell House reflects the field’s wide disciplinary reach, spanning natural product chemistry, pharmacological mechanisms, functional compounds, medicinal mushroom science, and translational biomedical research.

### 3.5. Most Relevant Institutions

The institutional distribution shows that bioactivity research on *G. lucidum* is strongly concentrated in Asian research systems, particularly China (Table 3). Shanghai Jiao Tong University ranked first with 69 publications, followed by the University of Malaya with 61, Nanjing Agricultural University with 59, Jiangnan University with 58, and the University of Belgrade with 55. The dominance of Chinese institutions reflects the location’s strong research capacity in medicinal fungi, natural product chemistry, pharmacology, biotechnology, and traditional medicine.

The presence of institutions from Malaysia, Serbia, and Taiwan indicates that the field also includes important regional and international contributors beyond mainland China. The University of Malaya and National Taiwan University highlight Southeast and East Asian engagement, while the University of Belgrade represents notable European participation. Overall, the table suggests that *G. lucidum* bioactivity research is institutionally anchored in locations with strong traditions in medicinal mushroom research, fungal biotechnology, ethnopharmacology, and bioactive compound discovery.

### 3.6. Scientific Co-Authorship and Collaboration Network

**Figure 7:**
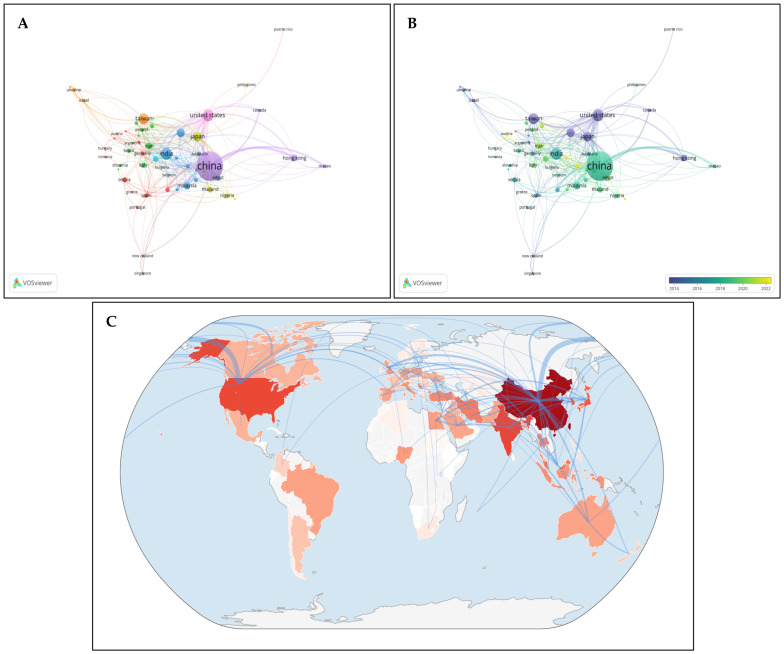
Co-authorship and collaboration patterns in bioactivity research on *G. lucidum* through (**A**) network analysis, (**B**) overlay visualization, and (**C**) collaboration world map.

The location-level co-authorship network shows that bioactivity research on *G. lucidum* is organized around a strongly connected but unevenly distributed international collaboration structure (Figure 7A). China occupies the largest and most central node, indicating its dominant role in both publication productivity and collaborative activity. Its dense linkages with the United States, Japan, India, Taiwan, Hong Kong, Malaysia, Thailand, and several European locations suggest that China functions as the principal hub of knowledge production and exchange in this field. The United States, Japan, India, and Taiwan also appear as important secondary nodes, serving as bridges between Asian research clusters and wider international networks.

The overlay visualization reveals a temporal shift in collaborative activity (Figure 7B). Earlier collaborations appear more strongly associated with established contributors such as the United States, Japan, Canada, and several European locations, whereas more recent activity is increasingly concentrated among Asian and emerging research contributors, including China, India, Malaysia, Thailand, Iran, and Nigeria. This pattern suggests that *G. lucidum* bioactivity research has progressively expanded from a relatively concentrated network of established pharmacology and natural product research systems into a more geographically diversified field. The recent prominence of Asian countries is consistent with the ethnomedicinal relevance, cultivation history, and growing biomedical interest in *G. lucidum* within the region.

The collaboration world map further demonstrates the global diffusion of *G. lucidum* bioactivity research, with strong transcontinental links radiating primarily from East and South Asia toward North America, Europe, the Middle East, and Oceania, as shown in Figure 7C. Countries with darker shading, particularly China, India, the United States, Japan, and several Southeast Asian partners, represent major contributors to the field, while curved links indicate active cross-border co-authorship. Although the map shows broad international participation, collaboration remains concentrated among a limited number of highly productive countries, with many regions in Africa, Latin America, and parts of Central Asia appearing less integrated into the global collaboration network.

### 3.7. Keyword Co-Occurrence Network Analysis

**Figure 8:**
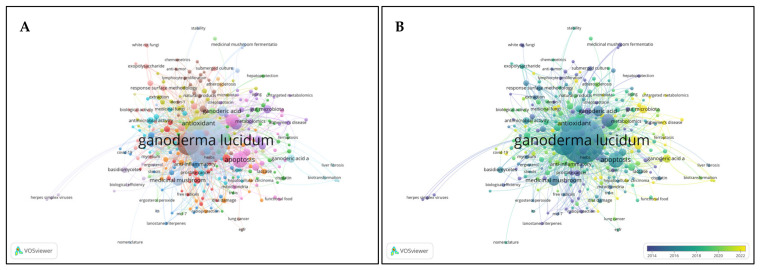
Keyword Co-occurrence through (**A**) network visualization and (**B**) overlay visualization of bioactivity research on *G. lucidum*.

The keyword co-occurrence network indicates that bioactivity research on *G. lucidum* is organized around a dense, highly interconnected thematic structure, with “*Ganoderma lucidum*” occupying the largest and most central node (Figure 8A). Closely linked high-frequency terms such as “antioxidant,” “apoptosis,” “anti-inflammatory,” “medicinal mushroom,” “ganoderic acid,” “metabolomics,” “gut microbiota,” “antimicrobial activity,” and “biological activity” show that the field is primarily anchored in pharmacological evaluation, bioactive compound characterization, and mechanism-oriented biomedical research. The prominence of “apoptosis,” “prostate cancer,” “lung cancer,” “hepatocellular carcinoma,” “mitochondria,” “DNA damage,” and “cisplatin” reflects a strong cancer biology orientation, whereas terms such as “free radicals,” “cardioprotection,” “hepatoprotection,” and “atherosclerosis” indicate sustained interest in oxidative stress-mediated protective effects.

The overlay visualization reveals a temporal transition in research emphasis (Figure 8B). Earlier studies appear more associated with general biological activity, extraction, antimicrobial activity, medicinal fungi, submerged culture, and polysaccharide-related terms, reflecting foundational work on cultivation, metabolite production, and preliminary bioactivity screening. More recent keywords, shown toward the yellow–green end of the overlay, include “gut microbiota,” “metabolomics,” “untargeted metabolomics,” “ferroptosis,” “Alzheimer’s disease,” “liver fibrosis,” “biotransformation,” and “functional food,” suggesting a shift toward systems-level profiling, disease-specific mechanisms, host–microbiome interactions, and translational applications. This pattern indicates that *G. lucidum* bioactivity research has evolved from broad pharmacological screening toward mechanistic, omics-driven, and application-oriented investigations.

### 3.8. Trend and Burning Topics

**Figure 9:**
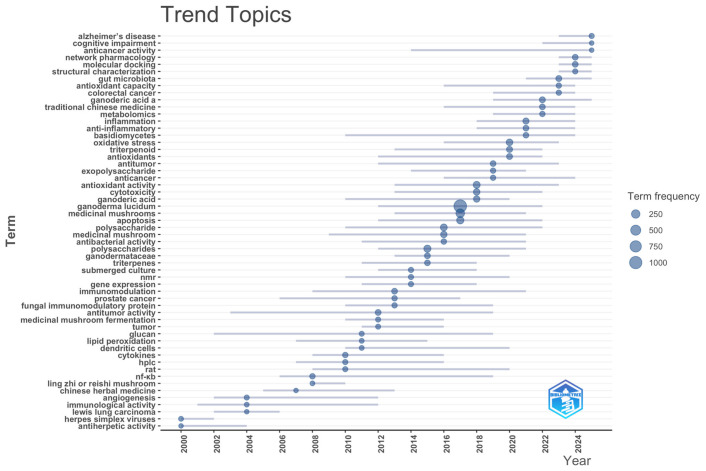
Topics studied over time in relation to bioactivity research on *G. lucidum.* Bubble size represents term frequency, classified into the following ranges: High (15–25 occurrences), Medium (8–14 occurrences), and Low (1–7 occurrences). The horizontal axis represents publication year, whereas the vertical axis lists the most frequently occurring research topics over time.

The trend-topic analysis shows a clear temporal progression from early antiviral and immunological themes toward broader pharmacological and mechanistic investigations of *G. lucidum* bioactivity (Figure 9). Around 2000–2005, early research attention was associated with terms such as “antiherpetic activity,” “herpes simplex viruses,” “immunological activity,” “angiogenesis,” and “lewis lung carcinoma,” indicating an initial focus on antiviral, anticancer, and immune-related bioassays. From approximately 2008 to 2015, the field expanded toward cellular and molecular mechanisms, as reflected by terms such as “NF-kB,” “cytokines,” “dendritic cells,” “lipid peroxidation,” “glucan,” “immunomodulation,” “gene expression,” “NMR,” “submerged culture,” “triterpenes,” and “ganodermataceae.” This period reflects a shift from general bioactivity screening to compound characterization, immunomodulatory mechanisms, and metabolite production systems.

From 2016 onward, the dominant themes became increasingly centered on high-frequency pharmacological and biomedical topics, including “*Ganoderma lucidum*,” “medicinal mushrooms,” “polysaccharide,” “apoptosis,” “ganoderic acid,” “cytotoxicity,” “antioxidant activity,” “anticancer,” “anti-inflammatory,” “oxidative stress,” and “basidiomycetes.” This thematic evolution parallels major scientific advances in the field. Earlier landmark investigations established polysaccharides as potent immunomodulatory macromolecules and triterpenoids as key contributors to the mushroom’s anticancer, anti-inflammatory, and antioxidant properties, thereby laying the conceptual foundation for mechanistic pharmacology. As high-throughput analytical technologies became widely available, research expanded beyond bioactivity screening toward metabolomics-based characterization of secondary metabolites, systems biology, and network pharmacology approaches that enabled more comprehensive elucidation of molecular targets and signaling pathways. More recently, growing recognition of host–microbiome interactions has driven increasing attention toward the role of *G. lucidum* polysaccharides in modulating gut microbial communities and their metabolites, particularly in relation to metabolic disorders, inflammatory diseases, neurodegeneration, and colorectal cancer. The emergence of keywords such as “metabolomics,” “gut microbiota,” “network pharmacology,” and “molecular docking” therefore reflects not merely changing terminology but a broader conceptual shift from descriptive pharmacological evaluation toward precision medicine, systems pharmacology, and translational biomedical research.

**Figure 10:**
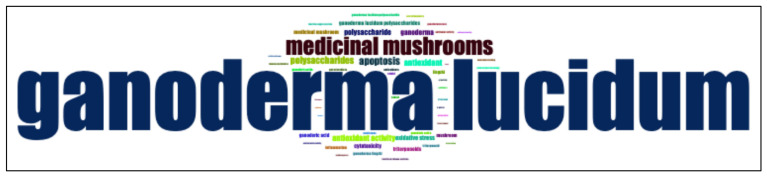
Word cloud visualizes the most frequently occurring terms in bioactivity research on *G. lucidum*, with themes in the field.

The word cloud shows that “*Ganoderma lucidum*” is the most dominant term, confirming that the dataset is strongly centered on this medicinal mushroom and its bioactivity-related literature (Figure 10). The frequent appearance of “medicinal mushrooms,” “polysaccharide,” “polysaccharides,” “ganoderic acid,” “ganoderma,” and “triterpenoid” indicates that the field is largely structured around the characterization of major bioactive constituents. These terms reflect the central importance of polysaccharides and triterpenoids as principal chemical classes responsible for the reported pharmacological activities of *G. lucidum*.

The occurrence of terms such as “apoptosis,” “antioxidant,” “antioxidant activity,” “anti-inflammatory,” “antitumor,” “cytotoxicity,” “oxidative stress,” and “inflammation” highlights the dominant biological themes in the field. These keywords suggest that bioactivity research on *G. lucidum* has focused heavily on cancer-related mechanisms, redox regulation, immune modulation, and inflammatory pathways. The presence of terms such as “biological activity,” “natural products,” “medicinal fungi,” and “extract” further indicates that the field remains closely linked to natural product pharmacology, functional food research, and therapeutic discovery.

### 3.9. Conceptual Structure Mapping

**Figure 11:**
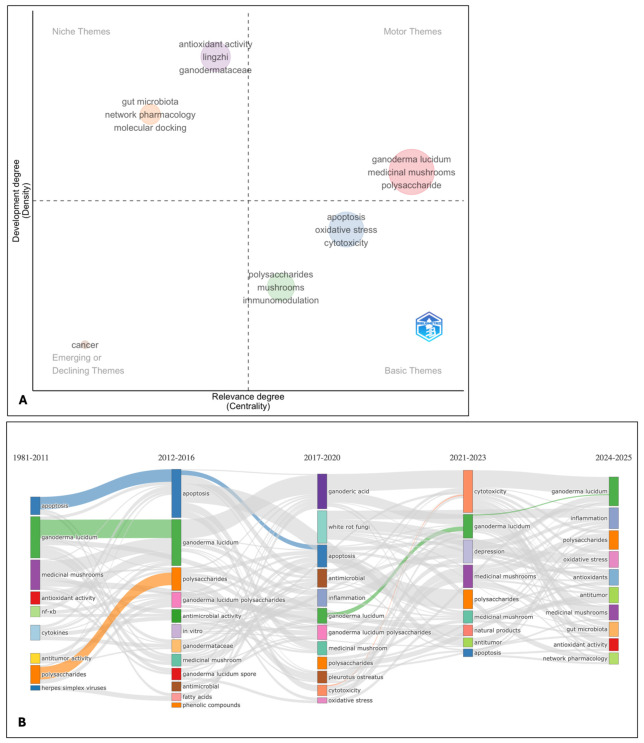
Strategic thematic map (**A**) and thematic evolution (**B**) of bioactivity research on *G. lucidum*.

The strategic thematic map shows that bioactivity research on *G. lucidum* is structured across themes with differing levels of centrality and development, as shown in Figure 11A. The basic themes quadrant contains “*Ganoderma lucidum*,” “medicinal mushrooms,” and “polysaccharide,” indicating that these topics are central to the field yet broadly connected to multiple research directions. Related themes such as “apoptosis,” “oxidative stress,” and “cytotoxicity” also appear in the basic-theme region, reflecting their importance as core mechanistic areas in anticancer and pharmacological studies. In contrast, “antioxidant activity,” “lingzhi,” and “ganodermataceae” appear as niche themes, suggesting specialized but internally developed areas, while “gut microbiota,” “network pharmacology,” and “molecular docking” represent emerging specialized themes linked to newer systems-level and computational approaches. The isolated position of “cancer” in the emerging or declining quadrant suggests that, as a standalone keyword, it may be less integrated than more specific mechanistic or disease-related terms.

The thematic evolution map demonstrates a clear transition from early broad pharmacological and immunological topics toward more mechanistic, compound-specific, and translational research themes (Figure 11B). During 1981–2011, the field was dominated by “*Ganoderma lucidum*,” “medicinal mushrooms,” “polysaccharides,” “antioxidant activity,” “NF-kB,” “cytokines,” “antitumor activity,” and “herpes simplex viruses,” reflecting early interest in immune modulation, antiviral effects, and general bioactivity screening. From 2012 to 2020, these themes evolved into more defined topics such as “apoptosis,” “ganoderic acid,” “*G. lucidum* polysaccharides,” “antimicrobial activity,” “in vitro,” “phenolic compounds,” “inflammation,” “cytotoxicity,” and “oxidative stress.” In 2021–2025, the field diversified further into “natural products,” “depression,” “gut microbiota,” “network pharmacology,” “inflammation,” “antioxidants,” “polysaccharides,” and “antitumor,” indicating increasing emphasis on disease-specific mechanisms, bioactive metabolite profiling, microbiome-related effects, and computational pharmacology.

**Figure 12:**
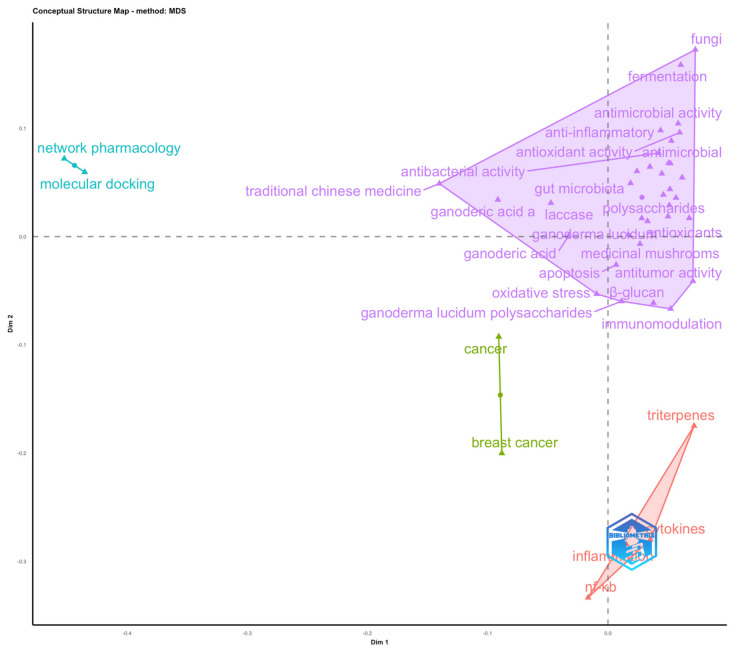
Factorial analysis through four-clustered multidimensional scaling (MDS) of bioactivity research on *G. lucidum*.

The factorial analysis through multidimensional scaling (MDS) reveals four distinct but interrelated conceptual clusters that collectively define the intellectual structure of bioactivity research on *G. lucidum* (Figure 12).

The largest cluster (purple) occupies the central region of the map and contains core terms such as “*Ganoderma lucidum*,” “medicinal mushrooms,” “polysaccharides,” “ganoderic acid,” “antioxidants,” “antioxidant activity,” “apoptosis,” “immunomodulation,” “β-glucan,” “gut microbiota,” “antimicrobial activity,” and “anti-inflammatory.” The proximity among these terms indicates strong thematic integration and frequent co-occurrence within the literature. This cluster reflects the dominant research focus of the field, encompassing the characterization of major bioactive compounds and their pharmacological activities, particularly antioxidant, immunomodulatory, anti-inflammatory, antimicrobial, and anticancer effects. The inclusion of “gut microbiota” and “traditional Chinese medicine” further suggests the integration of emerging systems-based and translational perspectives with the long-established medicinal use of *G. lucidum*.

A second cluster (red) comprises “triterpenes,” “cytokines,” “NF-kB,” and “inflammation,” representing a specialized mechanistic research domain focused on inflammatory signaling pathways and molecular immunology. The grouping of these terms indicates that studies within this cluster focus on elucidating how triterpenoids and related metabolites modulate cellular signaling networks involved in inflammation and immune regulation. The association of NF-kB and cytokines with inflammation highlights the importance of intracellular signaling cascades as targets for understanding the therapeutic mechanisms of *G. lucidum*. A third cluster (green), consisting primarily of “cancer” and “breast cancer,” forms a distinct disease-oriented thematic area. Its relative separation from the central cluster suggests that cancer-focused investigations constitute a specialized application domain that draws upon the broader pharmacological knowledge generated from studies of *G. lucidum* bioactive compounds.

The fourth cluster (cyan) contains “network pharmacology” and “molecular docking,” which are positioned separately from the main body of terms, indicating a conceptually distinct and emerging methodological domain. The close association between these two keywords reflects the increasing adoption of computational approaches for predicting molecular targets, identifying signaling pathways, and validating mechanisms of action associated with *G. lucidum* constituents. Their peripheral yet clearly defined placement suggests that computational pharmacology has become an important complementary framework to experimental bioactivity studies. Overall, the spatial arrangement of the four clusters demonstrates that *G. lucidum* bioactivity research has evolved into a multidisciplinary field integrating natural product chemistry, pharmacology, immunology, oncology, microbiome science, and computational biology, with strong connections between mechanistic investigations and translational biomedical applications.

## 4. Discussion

The scientometric findings demonstrate that bioactivity research on *G. lucidum* has evolved from a specialized area of medicinal mushroom research into a rapidly expanding and increasingly interdisciplinary scientific field. The sustained increase in publication output, particularly after 2010 and accelerating further after 2021, reflects growing global recognition of *G. lucidum* as a valuable source of pharmacologically active compounds. This trajectory is consistent with broader developments in natural product and medicinal mushroom research, where increasing attention has been directed toward bioactive polysaccharides, triterpenoids, proteins, sterols, and phenolic compounds with therapeutic potential [3,14]. The projected continuation of publication growth suggests that the field remains in an expansion phase rather than approaching intellectual saturation. Such growth is likely driven by the increasing demand for evidence-based nutraceuticals, functional foods, and complementary therapeutics, alongside advances in analytical chemistry, metabolomics, and molecular pharmacology that facilitate deeper characterization of fungal bioactive compounds [15,16]. The relatively stable citation performance observed since the early 2000s further indicates the development of a mature and interconnected knowledge base capable of sustaining continued scholarly influence despite the rapid increase in publication volume.

The overwhelming dominance of China across publication output, institutional productivity, authorship, and international collaboration reflects more than simple research capacity; it underscores the successful integration of traditional medicinal knowledge with contemporary biomedical research. Unlike many natural products that require rediscovery through modern screening approaches, *G. lucidum* has been used for centuries in traditional Chinese medicine as Lingzhi, providing an established therapeutic framework that has stimulated extensive scientific investigation [17]. The concentration of leading authors and institutions within China suggests the existence of highly specialized research ecosystems integrating fungal biotechnology, natural product chemistry, pharmacology, and translational medicine. Furthermore, the participation of countries such as Malaysia, India, Japan, Serbia, Iran, and the United States indicates that *G. lucidum* research has progressively transitioned from a regionally centered field into a globally distributed scientific enterprise. Nevertheless, the uneven geographical distribution of scientific production suggests persistent disparities in access to advanced analytical platforms, biotechnology infrastructure, and funding resources required for high-impact natural product research. Similar patterns of geographic concentration have been observed in other medicinal mushroom fields, where countries with strong ethnomedicinal traditions frequently emerge as dominant producers of scientific knowledge [18].

The co-authorship analyses reveal an increasingly interconnected international research network centered on China and supported by major scientific hubs across Asia, North America, and Europe. Such collaboration patterns are particularly important in medicinal mushroom research because comprehensive bioactivity investigations require expertise spanning taxonomy, cultivation technology, fermentation science, metabolomics, molecular biology, pharmacology, and bioinformatics. The temporal shift toward stronger participation by emerging Asian research systems suggests the globalization of fungal biotechnology and the broader accessibility of high-throughput analytical technologies. This expanding collaborative landscape appears to have facilitated the transition from descriptive bioactivity assessments toward mechanistic and translational investigations. At the same time, the continued concentration of collaborations among a limited number of highly productive countries indicates that knowledge production remains unevenly distributed. Increased inclusion of underrepresented regions may provide access to novel fungal strains, distinct cultivation practices, and diverse biogeographic resources that could further expand the pharmacological potential of *G. lucidum* and related medicinal fungi.

The thematic analyses collectively demonstrate a substantial transformation in the intellectual structure of research on *G. lucidum* bioactivity. Earlier studies focused primarily on broad pharmacological activities, including antiviral, antitumor, antioxidant, and immunomodulatory effects, reflecting a traditional natural product discovery paradigm centered on biological screening. The dominance of keywords such as polysaccharides, triterpenoids, apoptosis, oxidative stress, inflammation, cytokines, NF-κB, and immunomodulation indicates that contemporary research has moved beyond demonstrating efficacy toward elucidating the molecular and cellular mechanisms of action. This progression is supported by growing evidence showing that *G. lucidum* polysaccharides regulate immune cell activity, cytokine production, and macrophage function, while triterpenoids influence inflammatory signaling pathways, oxidative stress responses, and cancer-related molecular targets [19,20,21]. The sustained prominence of apoptosis, cytotoxicity, oxidative stress, and inflammation across thematic analyses further indicates that cancer biology and chronic inflammatory diseases remain central application domains for *G. lucidum* bioactive compounds. Moreover, the persistence of polysaccharide- and triterpenoid-related themes highlights the continued importance of these metabolites as the primary drivers of pharmacological interest in the species [22,23].

Perhaps the most notable development revealed by the bibliometric mapping is the emergence of systems-level and computational approaches. The appearance of keywords such as metabolomics, gut microbiota, network pharmacology, molecular docking, cognitive impairment, Alzheimer’s disease, and depression suggests a paradigm shift toward integrated systems biology and precision medicine frameworks. Recent studies have increasingly demonstrated that *G. lucidum* bioactive compounds exert therapeutic effects not only through direct molecular interactions but also through modulation of host–microbiome relationships and complex metabolic networks [3,24]. The emergence of gut microbiota and neurodegenerative disease themes reflects growing interest in the microbiota–gut–brain axis as a potential mechanism underlying the neurological and cognitive benefits attributed to *G. lucidum* [25,26]. Likewise, the distinct positioning of network pharmacology and molecular docking within the conceptual structure map suggests that computational approaches have become increasingly important for predicting molecular targets, elucidating signaling pathways, and prioritizing candidate bioactive compounds for experimental validation. Despite these advances, the literature remains dominated by in vitro and preclinical investigations, while comparatively fewer studies address pharmacokinetics, bioavailability, standardization, long-term safety, and clinical efficacy. Future progress in the field will therefore depend on strengthening the translational pathway from laboratory discovery to evidence-based therapeutic, nutraceutical, and functional food applications through the integration of multi-omics technologies, artificial intelligence-assisted drug discovery, and well-designed clinical studies.

## 5. Limitations of the Study

Several limitations should be considered when interpreting these findings. First, the analysis was limited to the Scopus database, which may underrepresent relevant publications indexed exclusively in other databases, including Web of Science, PubMed, Dimensions, or regional indexing platforms. As a result, some studies, especially those from developing countries or published in local journals, may not have been included. Second, the inclusion of only English-language publications may have excluded significant scientific contributions published in other languages, particularly from countries with established traditions in medicinal mushroom research. Third, bibliometric indicators, including publication counts and citation metrics, are affected by database coverage, publication age, journal visibility, and citation practices. Older publications typically have more opportunities to accumulate citations, which may introduce temporal citation bias. Additionally, countries, institutions, and authors with higher productivity may benefit from increased visibility and collaborative networks, potentially amplifying their bibliometric influence. Fourth, despite efforts to improve dataset accuracy through manual data cleaning and keyword harmonization, some inconsistencies in author names, institutional affiliations, and indexing terminology may remain and could affect network-based analyses. Finally, scientometric analyses assess patterns of scientific productivity, collaboration, citation impact, and thematic development, rather than the methodological quality, reproducibility, or clinical effectiveness of individual studies. Accordingly, the identified research hotspots, influential contributors, and emerging themes should be viewed as indicators of scholarly activity and knowledge evolution, not as direct measures of therapeutic efficacy or translational success. Despite these limitations, this study offers a comprehensive overview of the global development, intellectual structure, and emerging directions in bioactivity research on *G. lucidum*.

## 6. Conclusions

A scientometric analysis of 2877 publications indicates that bioactivity research on *G. lucidum* has exhibited sustained, accelerating growth over the past four decades, underscoring its growing significance in natural product science, pharmacology, biotechnology, and functional food research. Research output is primarily concentrated in Asian countries, particularly China, with international collaborations facilitating the field’s global expansion. Keyword, thematic, and conceptual analyses demonstrate a shift from broad bioactivity screening and compound characterization to mechanistic, disease-specific, and translational investigations, particularly those addressing apoptosis, oxidative stress, gut microbiota, metabolomics, network pharmacology, and molecular docking. Although significant scientific advances have been made, key translational challenges persist, including limited clinical validation, standardization challenges, and an inadequate understanding of bioavailability and long-term efficacy. The ongoing development of omics-based methodologies, microbiome research, and computational pharmacology indicates that *G. lucidum* is likely to remain a prominent source of bioactive compounds and therapeutic innovation. This study offers a comprehensive overview of the intellectual structure, research evolution, and prospective directions of *G. lucidum* bioactivity research, serving as a valuable reference for researchers, clinicians, and stakeholders in medicinal mushroom science.

## Figures and Tables

**Table 1:** Top 10 authors publishing on bioactivity research on *G. lucidum*.

Rank	Author Name	Affiliation	Location	TP
1st	Zhong, Jian-Jiang	Shanghai Jiao Tong University	China	47
2nd	Zhao, Ming Wen	Nanjing Agricultural University	China	38
3rd	Lin, Zhi-Bin	Peking University Health Science Center	China	37
4th	Ren, Ang	Nanjing Agricultural University	China	34
5th	Shi, Liang	Nanjing Agricultural University	China	33
6th	Zhang, Jing Song	Shanghai Academy of Agricultural Sciences	China	32
7th	Shimizu, Kuniyoshi	Kyushu University	Japan	31
8th	Xie, Yizhen	Guangdong Yuewei Edible Fungi Technology Co., Ltd.	China	30
9th	Janardhanan, Krishnan Krishnankutty	Amala Cancer Hospital and Research Centre	India	29
10th	Li, Zhen Hao	The Pharmaceutical Sciences of Zhejiang University	China	25

Abbreviation: TP: Total publication. Note: Rank was based on TP.

**Table 2:** Top 10 Scopus-indexed journals publishing research on the bioactivity of *G. lucidum*.

Rank	Journal Title	CiteScore 2025	Publisher	TP	TC	ACP	h-Index
1st	*International Journal of Medicinal Mushrooms*	3.3	Begell House	258	4734	18.35	31
2nd	*International Journal of Biological Macromolecules*	11.4	Elsevier	90	4304	47.82	37
3rd	*Journal of Ethnopharmacology*	11.2	Elsevier	59	2984	50.58	32
4th	*Molecules*	10.3	Multidisciplinary Digital Publishing Institute (MDPI)	41	986	24.05	18
5th	*Frontiers in Pharmacology*	9.5	Frontiers Media S.A.	40	1064	26.60	17
6th	*Carbohydrate Polymers*	23.1	Elsevier	36	3102	86.17	31
7th	*Scientific Reports*	6.4	Springer Nature	35	1789	51.11	21
8th	*PLOS One*	4.8	Public Library of Science	30	1496	49.87	22
9th	*Phytochemistry*	7.1	Elsevier	27	2837	105.07	20
10th	*Phytotherapy Research*	14.5	John Wiley & Sons	24	1192	49.67	18

Abbreviation: TP: Total publication; TC: Total citation; ACP: Average citation per paper. Note: Rank was based on TP.

**Table 3:** Top 10 institutions publishing research on bioactivity of *G. lucidum*.

Rank	Affiliation	Location	TP
1st	Shanghai Jiao Tong University	China	69
2nd	University of Malaya	Malaysia	61
3rd	Nanjing Agricultural University	China	59
4th	Jiangnan University	China	58
5th	University of Belgrade	Serbia	55
6th	China Medical University	China	54
7th	Fujian Agriculture and Forestry University	China	53
8th	National Taiwan University	Taiwan	51
9th	Zhejiang University	China	48
10th	Fudan University	China	47

Abbreviation: TP: Total publication. Note: Rank was based on TP.

## Data Availability

No new data were created or analyzed in this study. Data sharing is not applicable to this article.
